# Strengthening Community Participation by People With Disabilities in Community-Based Group Homes Through Innovative Action Research

**DOI:** 10.3389/fpubh.2022.747919

**Published:** 2022-04-29

**Authors:** Marji Erickson Warfield, Laura Lorenz, Hebatallah Naim Ali, Jody Hoffer Gittell

**Affiliations:** ^1^Center for Youth and Communities, Lurie Institute on Disability Policy, Heller School for Social Policy and Management, Brandeis University, Waltham, MA, United States; ^2^Heller School for Social Policy and Management, Brandeis University, Waltham, MA, United States; ^3^Institute for Health Systems, Heller School for Social Policy and Management, Brandeis University, Waltham, MA, United States

**Keywords:** community participation, residential facilities, long term care, relational coordination, action research, Olmstead decision, direct care staff, people with disabilities

## Abstract

In the US and beyond, a paradigm shift is underway toward community-based care, motivated by changes in policies, payment models and social norms. A significant aspect of this shift for disability activists and policy makers is ensuring participation in community life for individuals with disabilities living in residential homes. Despite a U.S. government ruling that encourages community participation and provides federal and state funding to realize it, little progress has been made. This study builds on and integrates the expanded model of value creation with relational coordination theory by investigating how the resources and relationships between care providers, adults with disabilities, family members, and community members can be leveraged to create value for residents through meaningful community participation. The purpose of our community case study was to assess and improve the quality of relationships between stakeholder groups, including direct care staff and managers, residents, family members, and the community through an action research intervention. This study took place in a residential group home in a Northeastern US community serving adults with disabilities from acquired brain injury. A pre-test post-test design was used and quantitative assessments of relational coordination were collected through electronic surveys, administered at baseline, and post-intervention. Direct care staff, supervisors, the house manager, and nursing staff completed the survey. Qualitative data were collected through focus groups, change team meetings, and key informant interviews. Direct care staff formed a change team to reflect on their baseline relational coordination data and identified the weak ties between direct care staff, family members, and the community as an area of concern. Staff chose to hold a community-wide open house to provide an opportunity to foster greater understanding among staff, residents, family, and community members. The change team and other staff members coordinated with local schools, business owners, town officials, churches, and neighbors. The event was attended by 50 people, about two-thirds from the community. Following the intervention, there was an increase in staff relational coordination with the community. While statistical significance could not be assessed, the change in staff RC with the community was considered qualitatively significant in that real connections were made with members of the community both directly and afterwards. Despite a small sample size, a residential setting where management was favorable to initiating staff-led interventions, and no comparison or control group, our small pilot study provides tentative evidence that engaging direct care staff in efforts to improve relational coordination with community members may succeed in building relationships that are essential to realizing the goal of greater participation in community life.

## Introduction

In the US and beyond, a paradigm shift is underway toward community-based care, motivated by changes in policies, payment models, and social norms. People with disabilities are being cared for in residential group homes rather than large institutional settings with the goal of enhancing their participation in community life (1). This shift to community living is allied with general principles of The Convention on the Rights of Persons with Disabilities (CRPD) that call for the full and effective participation and inclusion in society of all people.

In the US, the 1999 Olmstead decision, as part of the Americans with Disabilities Act (ADA), required that services for people with disabilities be provided in the most integrated settings possible (2). This was a landmark decision for disability rights, and states were required to develop Olmstead Plans to indicate how they would meet these requirements. An analysis of these plans, however, found that the main focus was on medical services and activities of daily maintenance, rather than on plans to involve residents in community planning and social activities that could facilitate their participation in community life (2, 3).

To more fully meet the Olmstead mandates, a broader paradigm shift from person-centered to relationship-centered care is required (4) through an expanded model of value co-creation across all those who care for the individual (i.e., care providers, family, etc.) and across different contexts (i.e., within the home and within the community where the home is located) (5, 6). Person-centered care means learning and supporting the values, preferences, and goals of care recipients and placing the care recipient at the center of dynamic relationships among his or her caregivers (7). Centering the care recipient enables their expertise and experiences to be a part of the process of providing high quality care (8). To fully achieve the Olmstead vision requires an expanded focus on the quality of relationships among all involved in the care process, including the caregivers, the care recipient, the family and members of the broader community (9). It is not sufficient to focus only on the micro-level (i.e., the environment within the home); a focus on community-level and societal-level factors that play a role in facilitating or limiting participation is also needed (10).

First-person accounts of what full community participation means to individuals with disabilities have informed policy discussions (10, 11). In addition to the physical aspects of community integration, individuals with disabilities care about the social and psychological aspects of community integration (12). A participant in the Angell et al. (11) study emphasized the importance of social connections: “It just feels better when you're being with people and being a part of something” (p. 5). Other first-person accounts note the importance of acceptance and casual connections to others in the neighborhood (13). Study participants have emphasized opportunities for social activities as a way of forming and maintaining social relationships (14). In our specific study context, three prior research projects found that residents consistently valued opportunities for social interaction both inside and outside their group home (15–17). One study used the visual action research method known as photovoice to increase awareness of environmental factors impacting the community integration of older adults with acquired brain injury (17). Residents have identified independence, relationships, and meaningful things to do as the key aspects of community integration and acknowledged that they require support from others to realize their community participation goals (17).

## Context and Aim

### Relational Coordination as an Approach to Strengthen Community Participation

Addressing these social and psychological aspects of community integration can be particularly challenging (10). Relational coordination theory deepens our understanding of relationship-centered care, its outcomes, and how it is achieved (18). Relational coordination is communicating and relating for the purpose of task integration and has been associated with a wide range of positive outcomes including quality and safety outcomes [e.g., (19, 20)], efficiency and financial outcomes [e.g., (21, 22)], staff well-being [e.g., (23, 24)], family well-being [e.g., (25)], and learning and innovation [e.g., (26)].

Research shows that many of these positive outcomes cannot be achieved by formal care providers alone (27). Relational coordination theory has thus expanded to include coordinating care with clients and their families, especially when care is delivered across multiple settings (28). Relational coordination between care providers and family members positively predicts care recipients' psychological well-being and clinical outcomes (28) and family members' quality of life (25). Relational coordination between care providers and care recipients has also been shown to predict the well-being of people with a range of care needs [e.g., (29)].

To integrate care recipients into the community, as mandated by the Olmstead Act, we expect that relational coordination may also need to be strengthened with the community. We know that direct care providers impact residents' quality of life (15) and their community participation (30). But there is little evidence regarding the strength of relational coordination between direct care providers and the community, and how to design interventions to strengthen coordination when needed. We expected that relational coordination might be relatively weak in the context of community-based residential care for people with disabilities due to some community members' discomfort with people who have disabilities (11). Differences in the ethnocultural background and language between staff of color and the communities in which they work may pose a further obstacle to relational coordination (31). Finally, when direct care staff are recent immigrants, ingrained cultural behaviors such as appropriate ways to interact with strangers may pose yet another obstacle to relational coordination (32).

In this paper, we describe a pilot study designed to assess and strengthen relational coordination between direct care staff and residents, families, and local communities. The work was guided by the Relational Model of Organizational Change which proposes that interventions can be designed to strengthen relational coordination among diverse stakeholders in order to achieve desired outcomes (33).

## Methods

### Setting and Population

The context for this study is residential care for people with disabilities associated with acquired brain injury (ABI). The benefits of community participation for people with chronic ABI include reduced mortality, slower rates of decline in cognition and physical function, lower drug use, reduced use of health services, and improved well-being (34). Since 2002 when the World Health Organization established a conceptual framework for functioning, disability and health (ICF) (35), the goal of community participation by people with disabilities has become a near-universal norm. Although the ICF conceptual framework encompasses both personal and environmental factors related to community participation, the impact of environmental factors on participation of people with disabilities in community-based group homes has received little attention.

### Study Design

The purpose of our community case study was to assess and improve relational coordination between stakeholder groups, including direct care staff and managers, residents, family members, and the community through an action research intervention. We utilized aspects of case study methodology tailored to program evaluation (36) and action research in health care settings (37). True to the action research approach, the project sought to investigate and improve practice through working collaboratively with staff to plan and evaluate new ideas and introduce innovations.

This pilot study used a pre-test post-test design in a community-based residential care site. Quantitative assessments of relational coordination were collected through electronic surveys, administered at baseline and post-intervention. Direct care staff, supervisors, the house manager, and nursing staff completed the survey. Qualitative data were collected through focus groups, change team meetings and key informant interviews to inform the change process and interpret quantitative findings. Direct care staff at the selected site were invited to form a change team to reflect on their baseline relational coordination data, identify areas of concern, and develop and implement an intervention to address them. The study protocol was approved by the Brandeis University Human Research Protection Program. In addition, the study protocol was also approved by the Research Review Committee at the Massachusetts Department of Developmental Services.

### Site Selection

Two of the four group homes for people with disabilities from acquired brain injury operated by a non-profit organization were considered for participation. To be eligible, a site had to meet four criteria: at least 2 years of operation, high quality of care as suggested by a lower staff turnover rate than the industry norm, full occupancy, and management willingness to support the study. Only two sites within the non-profit organization met all four criteria during the recruitment period. The paper will describe the study experience and community intervention developed in one of them. Although consideration was given to including the site that was not selected as a control or comparison site, the research team decided against this approach. Having a traditional control site would have required random assignment to two different sites which thwarts the participatory approach taken. In addition, looking at the second site as a comparison site would be comparing very different interventions making it hard to assess what additional insights would be gained. The second site identified a different problem to solve based on data gathered by the Relational Coordination (RC) survey. Staff discussion of those results yielded a different intervention.

The site selected was in a middle to upper-middle class suburban setting. Sixteen residents live in the home, which is located next to the community's downtown, including stores, coffee shops, the public library and near a small nature reserve with disability access. The residence was grandfathered from the current Medicaid requirement limiting group homes for individuals with ABI to four residents. Each resident has a private room and bathroom, shared dining and activity areas, and an adaptive exercise area on site.

### Recruitment

Following site selection, the research team conducted informational meetings with staff (i.e., direct care staff, supervisors, nursing staff, and managers) to introduce relational coordination concepts and extend an invitation to participate in the study. All staff received an email invitation to complete the baseline survey, as well as an invitation to participate on the change team. Surveys were administered online and de-identified by a third party to ensure anonymity. Staff members each received a $25 gift card upon survey completion. The rate of staff participation was 79% (15/19) at pre-test and 74% (14/19) at post-test.

The importance of ensuring that participation was voluntary informed the recruitment processes. Several approaches were taken to be clear that staff could participate or not (or participate occasionally) and that their decision regarding participation would not influence their job or future opportunities. All messaging about the project emphasized this. In addition, the fact that recruitment was continuous supported this message. Staff who did not participate in the early stages were invited again to participate as the project moved forward. There was an open invitation to participate throughout the life of the project.

A focus group was held to share baseline survey results with staff and gain their perspectives on those results. Facilitators described response rates and findings about relational coordination. The RC results indicated that timely communication and shared knowledge had the lowest scores, especially with the community. The focus group questions then asked: (1) What does community mean to you? (2) How does it feel to be in the community with residents? and (3) what can be done to change these experiences?

All members who attended the focus group were invited to join the change team. The change team's role was to discuss baseline results in-depth, define problems, and develop possible remedies to correct them. Change team meetings were held during the afternoon shift change to allow for greater participation and were facilitated by a research team member. Staff received a $15 gift card for each focus group or change team meeting attended.

### Sample

The sample was drawn from staff at the site. Two staff were in leadership positions, the house manager and a manager who oversaw several other residences for people with disabilities from acquired brain injury (ABI). Three-quarters of staff were fulltime, the rest were part-time and no one was employed casually. A total of 19 staff were employed when the project began and when it ended; 15 (79%) completed the baseline survey and 14 (74%) completed the post-intervention survey. Table 1 shows the response rate at baseline and post intervention by participant role. The direct care staff at the residence had worked there for 4 years on average and in residential care or human services for more than 6 years on average.

**Table 1:** Response rate by participant role.

**Participant role**	**Baseline (*n* = 15)^†^**	**Post-intervention (*n* = 14)**
Direct care staff	11 (73.3%)	11 (73.3%)
Program director	1 (100%)	1 (100%)
Program nurse	2 (100%)	2 (100%)
Residential supervisor	1 (100%)	0 (0%)

† *Response rate percentage were calculated using the total number of staff members eligible/invited (N = 19)*.

Socio-demographics of survey respondents at baseline (direct care staff, nursing staff, supervisors, and managers) are summarized in Table 2. Forty percent of respondents were female. Most of respondents had completed a bachelor's degree or higher and had worked in other professions in their countries of origin (e.g., accounting, education, social work, and music). Respondents had a median of 5 years of experience in direct care. About half of respondents were native English speakers from English-speaking countries in Africa. Three quarters of respondents worked full time.

**Table 2:** Staff demographics.

**Characteristics**	**Participants (*n* = 15)**	**(%)**
**Age**
18–44 years old	9	(60.0%)
45–64 years old	6	(40.0%)
**Gender**
Female	6	(40%)
Male	9	(60%)
**Education**
Associate degree or less	8	(53.3%)
Bachelor's degree or higher	7	(46.7%)
**Management** ^a^
Yes	4	(26.7%)
No	11	(73.3%)
Experience in years: Median [range]	5.0	[1–21]
**English as first language**
Yes	7	(46.67%)
No	8	(53.3%)
**Working full-time**
Yes	11	(73.33%)
No	4	(26.67%)

*Demographics are based on staff responses to the baseline surveys*.

a *Management includes: program director, program manager, residential supervisor, and senior direct care workers workgroups*.

For the intervention (change team) meetings described in detail below, seven direct care staff and the House Manager participated in all of the meetings. One direct care staff member and the House Manager participated in every change team meeting. Three direct care staff members participated in almost all the meetings; each missed one meeting. Three direct care staff participated 1–2 times each closer to the end of the project when planning for the event was being finalized.

### Intervention

The intervention comprised two main components. The first component was a rigorous discussion of baseline relational coordination survey results among members of the change team. The second component was the development and implementation of a participatory event to strengthen the weakest ties identified by the relational coordination survey—with family members and community members.

The development of the participatory event was an extension of earlier research at the site and therapeutic practices utilized there. In an earlier qualitative study, residents expressed interest in involvement in the community (15), which is also an expressed goal of residents' family members and guardians. For this and all studies conducted at the site, family members and guardians are required to provide informed consent for participation of their loved one, and residents provided informed assent. In the Northeastern US where this study took place, it is common practice for people living with ABI to “tell their stories” during support group meetings and brain injury prevention activities in schools, in the community, and at policy advocacy events. In the study setting, activities creating art and songs about their lives, and research using photographs and captions sharing residents' perspectives about their community integration (17) provide valued opportunities to be “seen and heard.”

A major activity planned for the participatory event was a “fishbowl” exercise. Change team members thought this would be an effective way to engage event participants. Change team members developed the questions to be answered in the fishbowl. Residents were asked the questions verbally. For other groups questions were provided on index cards. Questions for residents included: How did you get your brain injury? How did it change your life? How do staff help you? How do they help you reach your goals? For staff, questions included: What was your work before you moved to America? What is your experience working in brain injury? How has it changed over time? How do you feel about working at this residence? What supports and challenges do residents and staff find in the community? Do you have anything else you would like to say? For family and community members, questions included: What is your view of brain injury? How does this residence contribute to the community? How can you contribute to life at the residence? How can you help to increase participation in the community by residents and care staff? What have you learned today?

### Data and Measures

The validated relational coordination survey (38–40) was completed by staff pre- and post-intervention. The survey includes seven dimensions—frequency, timeliness, accuracy, and problem-solving nature of communication, and the extent to which relationships are characterized by shared goals, shared knowledge, and mutual respect—which together form a construct called relational coordination or RC, with scores ranging from 1 to 5, where higher scores indicate stronger relational coordination.

Relational coordination is typically measured from the perspective of each key role in the work process—in this case staff, residents, family members, and community members—allowing the creation of a complete network measure (41). As recommended in the case of data limitations, we measured only the staff's experience of relational coordination with each of the other key roles—residents, family members, and community members. Averaging together the seven dimensions of relational coordination for each of the roles, we constructed three separate measures: staff RC with residents, staff RC with family members, and staff RC with community members. Higher scores represent stronger relational coordination with residents, family members and community members, from the perspective of staff. The reliability coefficient (Cronbach's alpha) for RC with residents was 0.72 at baseline and 0.67 post-intervention, RC with family members was 0.77 at baseline and 0.73 post-intervention, and RC with community members was 0.90 at baseline and 0.80 post-intervention.

Data were also qualitative and included participant observation notes, notes recorded on flip charts during focus group discussions and change team meetings, and transcripts from key informant interviews. Change team meetings were not audio-recorded due to the potential to re-traumatize or cause discomfort for direct care staff who had migrated from their home countries in Africa due to civil unrest or authoritarian regimes. A research assistant (RA) took extensive notes during the meetings and wrote significant statements verbatim. A co-Principal Investigator (co-PI), the second author, reviewed, and added to the RA's notes after each meeting.

### Data Analysis

Descriptive statistics were generated to characterize survey results. RC data were plotted at baseline and post-intervention. The quantitative data analysis plan was centered on examining pre and post intervention differences based on the data from the validated RC survey.

Qualitative data included: (1) focus group discussions on the baseline RC survey results and the post-intervention RC survey results, (2) change team meeting notes, and (3) post-intervention interviews with the House Manager, a Senior direct care staff member, and a change team member. These data were used to reflect back to participants and inform the change process and were analyzed to identify themes and quotes (42) that enhanced the description of the residential home and understanding of the direct care staff experience. Direct quotes were captured to illustrate the emerging themes. The initial thematic analysis was done by the RA. The co-PI separately reviewed a sample of the notes. The RA and co-PI discussed their findings and adjusted the thematic analysis as needed to reach consensus.

The mixed methods data analysis—integrating the quantitative and qualitative data—was based on the approach of blending variables and themes as described by Creamer (43) to develop a fuller understanding of the phenomenon being examined.

## Results

### Baseline Data

The residential home had been designed to limit solitude and isolation and to provide a community-integrated residential alternative for people with disabilities from acquired brain injury. Yet at baseline, staff reported their weakest relational coordination ties with the community (3.10 on a 5-point scale), followed by somewhat stronger ties with families (3.55 on a 5-point scale). Poor relational coordination between staff and community members is reflected in selected quotes in Table 3 (excerpts #1, 3, 4). Reflecting on the data, staff attributed their weak ties with the community to racial and disability stigma, as reflected in these quotes:

**Table 3:** Themes, definitions, subthemes, excerpts, and sources: sample qualitative data findings.

**No**.	**Themes and sub-themes**	**Definitions**	**Quote or excerpt from qualitative data source**	**Source**
	**Knowledge**	**Knowledge among community, family, and DCS**		
1	Brain injury	Impact of injury on cognition, behavior, and lives	The community needs to have greater awareness of brain injury, that it could happen to anyone, that it should not be stigmatized.	FGD 1-19-17
2	DCS role and work	Role and care work of DCS	The community does not know who we are. How can we get to know each other? How can we get them to accept us?	CTM 1-26-17
	**Community**	**Local community (residents, businesses, etc.)**		
3	Stigma	Stigma toward residents and care staff	The community does not understand that brain injured individuals are not harmful, despite some potential behavioral issues.	CTM 3-16-17
4	Respect	Respectful treatment and perceptions (or lack of)	Our work with residents who have brain injury is not valued.	CTM 1-26-17
5	Sustainability	Sustainability of efforts to engage community	Building relationships between the community and residents and staff is a cycle. This will be an ongoing process.	CTM 3-16-17
	**Family**	**Family members of group home residents**		
6	Respect	Respectful interactions (or lack of same)	Negative behaviors by family hurt staff morale and make staff feel their care work is not appreciated.	CTM 2-23-17
7			Some family members are surprised to learn that “staff really care.” This new information makes them want to know staff better.	CTM 3-2-17
8	DCS role and work	Role and care work of DCS	Yelling at staff by family members shows lack of respect for staff role	CTM 2-23-17
	**Communication**	**Communication between community and DCS, and family and DCS**		
9	Sustainability	Build new communication skills	Staff can be supported by management to learn ways to curb rude behavior from family. For example, the rudeness of others can be limited with polite talk, e.g., “How are you today?”	CTM 3-16-17
10			We are concerned about how our comments might be received by the community and family. We need advice on communication and language.	CTM 3-31-17
11	Respect	Respectful interactions (or lack of same)	We need to thank the community and make sure they know how much we appreciate their presence and what they already do.	CTM 3-31-17
12			I felt people were listening.	SM 4-21-17
	**Intervention**	**Community event planned by change team**		
13	Goal	Goal for intervention	Improve relationships between residents and community, staff and family, staff, and community.	CTM 1-26-17
14	Activity	Activity at intervention	A fishbowl exercise is better than a panel presentation because it is informal, and more people can participate.	CTM 3-9-17
15			We want to share the fact that the US and English-speaking African countries were colonized by England, and each has resistance heroes (like the Minutemen).	CTM 3-9-17
16	Outcome	Self-report, during FGD or KII	An expectation has been set: staff are going into the community, and the community has said to us “Come to us, we will be welcoming.”	KII June 2017 (DCS)
17			The level of effort involved in a project like this is a barrier. Keeping staff involved is difficult.	KII June 2017 (HM)

*CTM, change team meeting; DCS, direct care staff; FGD, focus group discussion; HM, house manager; KII, key informant interviews; SM, staff meeting*.

*People are not friendly. They have faces of “stone.” This is hurtful for staff and for residents* (FGD 1-19-17).

*There is always the anxiety of drop in houses prices with a disability residence facility in the neighborhood. They won't encourage our presence by bonding with us or the residents* (FGD 1-19-17).

*Being black [in America] is often associated with crime. People feel afraid to come say hi, when we are pushing [or] assisting the residents in the neighborhood* (CTM 3-16-17).

#### Intervention Developed

Direct care staff and members of the research team formed a change team to address these issues. The action research principals of working collaboratively, evaluating new ideas, and trying something innovative (37) were utilized throughout the change team's work. The change team met 10 times over a 6-month period, with 3–4 workers participating consistently and one who became a champion, sharing information among all staff, and encouraging participation in the intervention. Change team members were slow at first to take on assignments that required attention during non-work hours. Finding an entry point was a key to engagement. Identifying a commonality in the U.S.-African history of colonization and resistance helped to motivate participation as did highlighting staff efforts to improve residents' quality of life (see Table 3, excerpt #15).

To address weak ties with the community, staff chose to hold a community-wide open house to provide an opportunity to foster greater understanding among staff, residents, family, and community members. The change team and other staff members coordinated with local schools, business owners, town selectmen, churches, and neighbors. The communication that occurred in preparation for the Open House was the first communication to occur between the home's staff and some family members and members of the community (see Table 3, excerpts #2, 7). The agenda for the Open House evolved to address (1) the causes and effects of brain injury among residents, their prior and current lives, and their perceptions of direct care staff, (2) the motivations, backgrounds, and culture of origin of the direct care staff, and (3) ways that community members could better support residents' community participation (see Table 3, excerpts #1, 2, 3, 4, 10). The agenda included fishbowl presentations by residents (their stories), direct care staff (their work and personal stories), and family and community members (their hopes for their loved ones, their perceptions of care provided, and their efforts to encourage community participation). The event was attended by 50 people, about two-thirds from the community, including elected officials, business owners, members of a local church, high school students, volunteers, and neighboring homeowners (see Table 3, excerpt #12).

The event started an exchange of perspectives and opened discussion on issues such as lack of access to local businesses due in part to uneven sidewalks. Family members who attended expressed appreciation for staff's dedication to providing care for their loved ones, a “new” viewpoint for some family members. The intervention was the entire process of engagement between staff, residents, family members, and community members described in this section, and not just the event itself. The process of engagement was expected to strengthen relational coordination between staff members and key community stakeholders, and also family members. It was seen as a beginning of ongoing efforts (see Table 3, excerpts #5, 7, 12, 13).

### Outcomes

**Figure 1:**
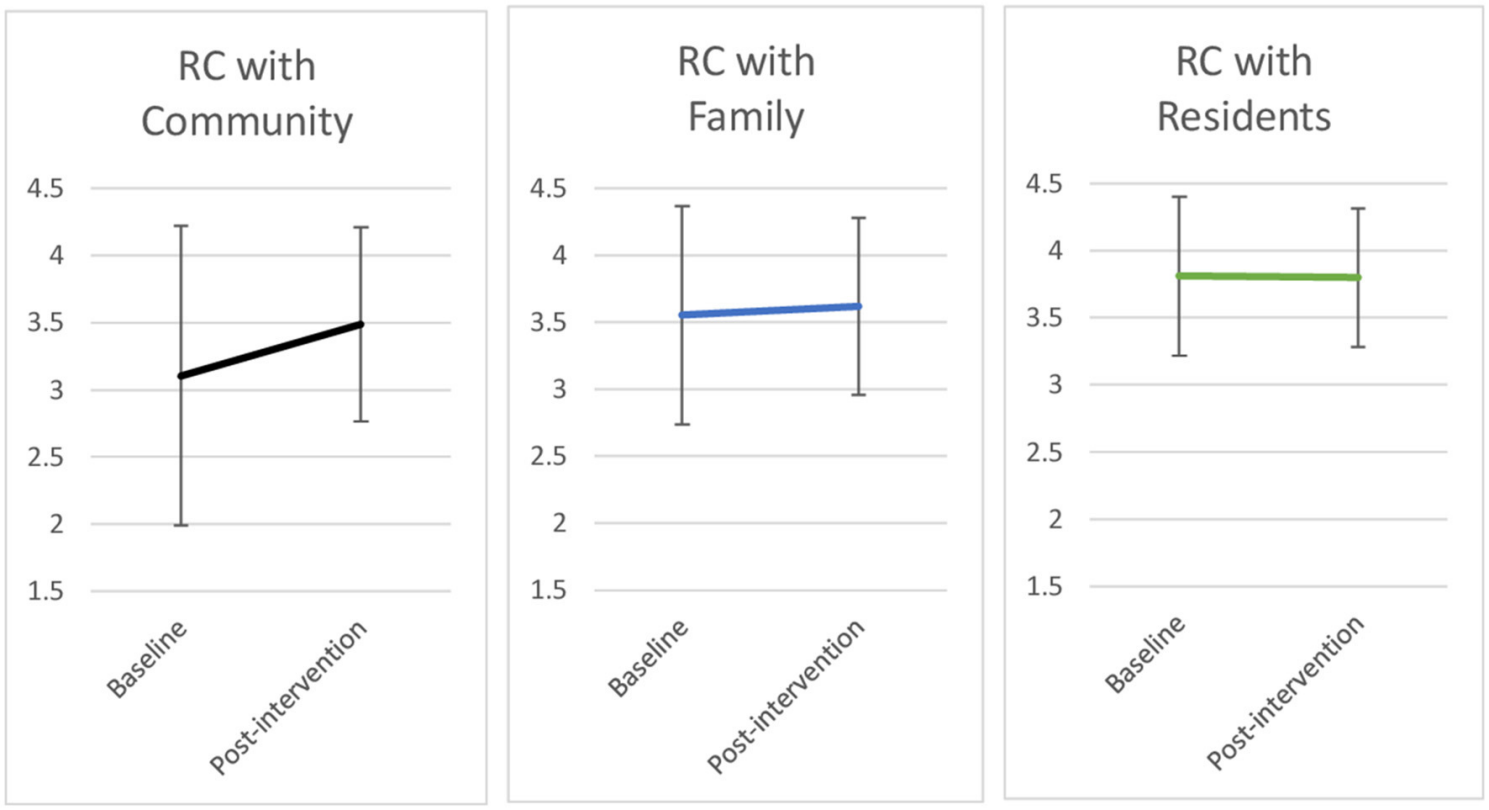
Changes in relational coordination with the community, family, and residents^†^.^†^Line graph shows changes in RC scores at baseline and post-intervention and their standard deviation. Sample sizes: baseline (*n* = 15); post-intervention (*n* = 14).

Changes in RC as experienced by direct care staff are shown in Figure 1, which plots the mean scores for each RC index using data gathered at baseline and post intervention. The mean change between each data point is represented by the line connecting the points for each measure.

Following the intervention, staff RC with the community increased by 0.38 of a point, while RC with families (+0.07) and residents (−0.01) remained relatively constant. While statistical significance could not be assessed, the change in staff RC with the community was considered qualitatively significant in that real connections were made with members of the community as a result of the intervention both directly and afterwards (see Table 3, excerpts #12, 16).

To assess what has happened since the community event took place, the house manager was interviewed by two of the research team members. The focus of the interview was to identify events and experiences enjoyed by residents and staff that provide evidence of greater community participation after the open house event. Activities such as a staff appreciation event at a local church and a weekly “Let's Eat Together” program orchestrated by the town on Wednesday evenings are examples of the value co-created by the action research intervention. Unfortunately, these have since been disrupted due to COVID-19. The interviews highlighted the challenge of sustaining this type of participatory action effort by direct care staff and their managers (see Table 3, excerpts #5, 17).

## Discussion

Even though state and federal funders have sought to increase participation in community life by people with disabilities, a focus on safety and assistance with Activities of Daily Living has dominated the residential care work environment (44). Realizing the goal of greater participation in community life may require a greater focus on building relationships with community members.

Our study identified weaknesses in relational coordination especially between direct care staff and the local community. These findings may have been due to cultural differences between immigrant staff and the families they serve and the communities in which they were embedded, and by negative family and community stereotypes toward both staff of color and people with disabilities (45) (see Table 3, excerpts #4, 6, 8). Results suggest that interventions may have the potential to support the development of positive relationships between people with disabilities in community group homes and the broader communities in which these homes are located. For this to happen, however, cultural barriers toward people with disabilities and foreign-born direct care staff, including ableism and racism, may need to be addressed through relational interventions as they were in this pilot study (see Table 3, excerpt #9).

### Feasibility of Participatory Intervention Processes

Our study suggests that it is feasible to engage direct care staff in designing and implementing interventions to strengthen relational coordination with key stakeholders. Our participatory intervention process—the use of change teams, surveys, and a co-created intervention based on the Relational Model of Organizational Change—provided direct care staff with opportunities to share their experiences of their work, their work environment and the meaning of their work. These opportunities resulted in workers feeling heard. The participatory intervention methods used in this study may thus promote relational coordination where there is organizational support to address the problems identified (46). Long-term sustainability of these types of efforts can be challenging (see Table 3, excerpt #5, 17).

### Involving Family and Community Stakeholders in Interventions

Study findings suggest that improving relational coordination in residential care means expanding our understanding of the caregiving role to include family members and community members, and taking concrete steps to support direct care staff in playing a bridging role between residents, their families and their communities. We suggest calling this conceptual expansion “relationship-centered care” to recognize the broader set of relationships that can contribute to quality of care and quality of life for people with disabilities living in the community. Adopting a relationship-centered care approach could help direct care staff, family members, and community members appreciate the interconnectedness of the care process for people with disabilities in community settings and achieve better quality of life and outcomes for them (47).

### Supporting Relationship-Centered Care Through Human Resource Management

The one-time event implemented during the study was not expected to alter the status quo permanently, but rather to initiate ongoing efforts to build high quality relationships among direct care staff, families and the community (see Table 3, excerpt #5). Such efforts will require ongoing leadership support, including changes in human resource practices as suggested by relational coordination theory [e.g., (38)]. An essential next step is therefore to support the bridging role of direct care staff through job design and other supporting human resource practices (48). Revising human resource practices to hire and support individuals who are willing to interact with family members and community members may be essential (32). Revised job descriptions, training and performance evaluations for direct care staff could all support engagement with family and community. When staff come from cultures that are different from that of residents, their families and the local community, additional support could include training in ways of communicating with people in the community when accompanying residents to activities. This training could focus on how the staff can use polite language as well as how they can handle conversations when family or community members are rude (see Table 3, excerpts #9, 11). Residential managers could also use a relational coordination framework when orienting family members of new residents to help families understand the important role that direct care staff play in helping residents to achieve the community integration they desire, with coaching for how they as family members can help. Providing a foundation of stronger relational coordination inside and outside the residence requires consistent messaging internally and externally that community-based residential care is best provided by a coordinated network of individuals and roles, including direct care staff, family members, and community members.

Our study recommendations are consistent with relational coordination theory and a growing number of studies in multiple sectors showing that human resource practices are a significant driver of relational coordination and associated performance outcomes, for better or worse depending on their design [e.g., (22, 38, 49–51)]. Our recommendations also build on our findings that it may be possible to intentionally improve relationships between direct care staff and the community through low cost, replicable interventions.

### Limitations and Challenges

Although the findings from this pilot test of innovative interventions by direct care staff are consistent with an emerging evidence base regarding relationship-centered approaches to care, our sample was small with insufficient observations to assess statistical significance of changes in relational coordination. No power analysis was conducted a priori to calculate a desired sample size. Maximizing the sample size was limited by the number of staff members working at the residence.

Second, we selected a site that already had management support for the change process and low staff turnover. Our selection protocol facilitated the success of our pilot study but also introduced the challenge of generalizing findings beyond high-functioning sites. The ability to generalize the findings is also influenced by the fact that staff were remunerated for their participation in data collection and the change team. Third, our pilot measured relational coordination with multiple stakeholders from the perspective of staff only, as in a prior influential study of residential care (22). Follow up studies would benefit from assessing relational coordination from the perspective of residents, family members, and community members.

Fourth, the research assistant (RA) and co-PI who participated in the change team meetings reflected together on their researcher lenses. The RA was a graduate student from Africa (Arabic-speaking North Africa). The co-PI had lived and worked for 4 years in two different sub-Saharan African countries and had traveled for shorter assignments to another six African countries. The researchers reflected that their life and work experience likely generated in them a greater level of empathy and understanding with regard to the project's change team members as compared to the average US researcher who had not experienced life and work in Africa. In particular, they could appreciate that direct care staff were educated professionals with a certain status in their home countries, and that they had changed their status when they emigrated to the US and started working in direct care for people with brain injury. Their researchers' lenses could be considered a strength of this pilot project.

The endeavor was designed to be a small pilot case study, with a minimal budget and a short- timeline. Thus, we focused on engaging staff with the hope of learning from them and building relationships with them as starting points for including the perspective of residents, family members, and community members in the future.

## Conclusion

Our small pilot study provides initial, tentative evidence that engaging direct care staff in efforts to improve relational coordination with residents, family members, and community members may succeed in building relationships that are essential to realizing relationship-centered care. Community-based residential care will continue to grow as a policy-mandated alternative to institutional care for people with disabilities. In this context, finding ways to improve relationships among staff, residents, family members, and community members becomes increasingly urgent. Engaging direct care staff in data-driven efforts to improve relationship-centered care for people with disabilities living in the community is one potential solution.

## Data Availability Statement

The raw data supporting the conclusions of this article will be made available by the authors, without undue reservation.

## Ethics Statement

The studies involving human participants were reviewed and approved by Brandeis University Human Research Protection Program and the Research Review Committee, Department of Developmental Services, Massachusetts. The patients/participants provided their written informed consent to participate in this study.

## Author Contributions

All authors listed have made a substantial, direct, and intellectual contribution to the work and approved it for publication.

## Funding

This research was funded by a grant from Brandeis University's Provost Office to JG.

## Conflict of Interest

The authors declare that the research was conducted in the absence of any commercial or financial relationships that could be construed as a potential conflict of interest.

## Publisher's Note

All claims expressed in this article are solely those of the authors and do not necessarily represent those of their affiliated organizations, or those of the publisher, the editors and the reviewers. Any product that may be evaluated in this article, or claim that may be made by its manufacturer, is not guaranteed or endorsed by the publisher.
